# Methyl donor S-adenosylmethionine (SAM) supplementation attenuates breast cancer growth, invasion, and metastasis *in vivo*; therapeutic and chemopreventive applications

**DOI:** 10.18632/oncotarget.23704

**Published:** 2017-12-26

**Authors:** Niaz Mahmood, David Cheishvili, Ani Arakelian, Imrana Tanvir, Haseeb Ahmed Khan, Anne-Sophie Pépin, Moshe Szyf, Shafaat A. Rabbani

**Affiliations:** ^1^ Department of Medicine, McGill University Health Centre, Montréal, Canada; ^2^ Department of Pharmacology and Therapeutics, McGill University, Montréal, Canada; ^3^ Department of Pathology, Fatima Memorial Hospital, Lahore, Pakistan

**Keywords:** DNA methylation, breast cancer, metastasis, SAM, epigenetics

## Abstract

DNA hypomethylation coordinately targets various signaling pathways involved in tumor growth and metastasis. At present, there are no approved therapeutic modalities that target hypomethylation. In this regard, we examined the therapeutic plausibility of using universal methyl group donor S-adenosylmethionine (SAM) to block breast cancer development, growth, and metastasis through a series of studies *in vitro* using two different human breast cancer cell lines (MDA-MB-231 and Hs578T) and *in vivo* using an MDA-MB-231 xenograft model of breast cancer. We found that SAM treatment caused a significant dose-dependent decrease in cell proliferation, invasion, migration, anchorage-independent growth and increased apoptosis *in vitro*. These results were recapitulated *in vivo* where oral administration of SAM reduced tumor volume and metastasis in green fluorescent protein (GFP)-tagged MDA-MB-231 xenograft model. Gene expression analyses validated the ability of SAM to decrease the expression of several key genes implicated in cancer progression and metastasis in both cell lines and breast tumor xenografts. SAM was found to be bioavailable in the serum of experimental animals as determined by enzyme-linked immunosorbent assay and no notable adverse side effects were seen including any change in animal behavior. The results of this study provide compelling evidence to evaluate the therapeutic potential of methylating agents like SAM in patients with breast cancer to reduce cancer-associated morbidity and mortality.

## INTRODUCTION

Despite the advancements being made in our understanding of the biology, diagnosis, prevention, and treatment of cancer, metastasis remains the dominant cause of breast cancer-associated morbidity and mortality [1]. The 10-year survival rate for stage I/II breast cancer patients, whose cancer is localized within the breast tissue, is around 88% [2]. However, the 10-year survival rate for Stage III and IV cancer patients with metastatic spread of breast tumors is 40% and less than 10% respectively [2]. Hence, there is an urgent need for the development of novel and less toxic therapeutic strategies that can be useful to block both tumor growth and metastatic spread of cancer cells.

Tumor metastasis occurs when the cancer cells are dislodged from the primary site due to their ability to degrade the component of the extracellular matrix, invade into the blood vessels through intravasation, survive in the circulation, extravasate from the blood vessels, and finally start to proliferate as new tumors at a distant organ [3]. The highly-organized multi-step process of metastasis is regulated and driven by networks of growth factors, cytokines, adhesion molecules, and proteolytic enzymes [4]. We and others have shown that several key molecules implicated in the metastatic cascade are epigenetically regulated through DNA hypomethylation [5–7]. For example, a positive correlation between promoter hypomethylation and subsequent increase in the expression of protease-encoding urokinase plasminogen activator (*PLAU*) gene has been observed with the progression of breast and prostate cancer [8, 9]. Some other cancer-related genes that are induced by DNA hypomethylation include heparanase (*HPSE*) [10], *synuclein-γ* (*SNCG*) [11], pro-opiomelanocortin (*POMC*) [12], cadherin 3 (*CDH3*) [13], related RAS viral oncogene homolog (*R-RAS*) [14], *maspin* (also called *SERPINB5*) [15], and S100 calcium binding protein P (*S100P*) [15]. Moreover, pharmacological inhibition of methylation of non-invasive breast cancer cell lines (MCF-7, ZR-75-1) by using 5-Aza-2´-deoxycytidine increased the expression of prometastatic genes like *PLAU*, HPSE, C-X-C motif chemokine receptor 4 (*CXCR4*), and *SNCG*, and thereby transformed them into more invasive cells [16]. Therefore, it stands to reason that the use of inhibitors targeting hypomethylation to downregulate genes of the metastatic cascade may serve as a suitable anti-cancer therapeutic strategy.

The universal methyl donor SAM (also known as AdoMet) could be used in this regard as an inhibitor of demethylation/hypomethylation. SAM is a naturally occurring physiologic molecule found ubiquitously in all living cells, and functions in transmethylation, transsulfuration, and aminopropylation pathways [17]. SAM is second only to adenosine triphosphate (ATP) in terms of playing a versatile role in different types of physiological processes [18]. Currently, it is used as a preventive agent for mood disorders, fibromyalgia, and joint pain. Even though the chemical structure of SAM was first described in the 1950s by Cantoni [19], its potential use as an anti-cancer therapeutic agent has only emerged over the last two decades [20]. SAM-treatment has been found to be effective in repressing the invasiveness as well as proliferative capabilities of different types of cancer cell lines [21, 22]. We have previously shown that the anti-metastatic activity of SAM is likely due to downregulation of pro-metastatic genes like *PLAU* and matrix metalloproteinase 2 (*MMP2)* [6, 23]. SAM has been shown to inhibit angiogenesis [24], and reduce inflammation-induced colon cancer [25]. Taken together, these studies provided a strong rationale towards the possible use of SAM in cancer prevention and treatment. However, the anti-cancer effects of SAM have never been examined in a therapeutic setting for hormone-dependent malignancies like breast cancer.

In the present study, we have investigated whether blocking demethylation and promoting methylation by SAM-treatment alone could exhibit anti-tumor effects using well-established *in vitro* and *in vivo* models of breast cancer. Results from this study show that SAM-treatment causes a significant reduction in tumorigenesis and metastatic spread of breast cancer cells which can be attributed in part to the ability of SAM to impact methylation and downregulation of the expression of several important genes implicated in the metastatic cascade.

## RESULTS

### SAM-treatment suppresses cell proliferation, migration, invasion, anchorage-independent growth and potentiates apoptosis *in vitro*

**Figure 1:**
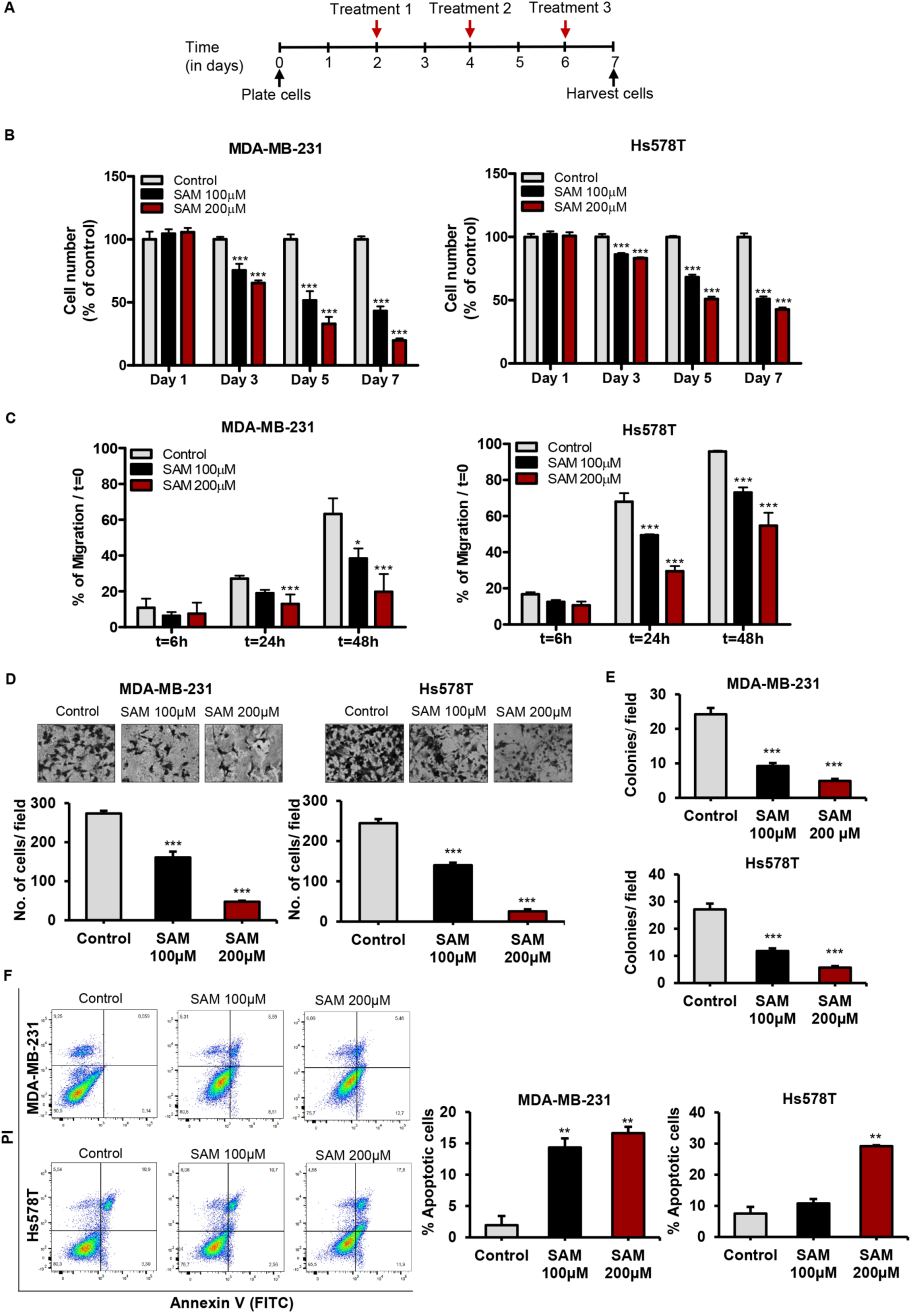
Effect of S-adenosylmethionine (SAM) on breast cancer cell proliferation, migration, invasion, anchorage-independent growth, and apoptosis *in vitro* **(A)** Schematic diagram of the treatment strategy for all the *in vitro* experiments. Human breast cancer cells MDA-MB-231 and Hs578T were treated with SAM (100 and 200 μM) by directly adding it to regular growth medium every other day from day 2 until they were harvested. **(B)** Human breast cancer cells MDA-MB-231 and Hs578T were plated in 6-well plates and treated with vehicle alone as control or SAM (100 and 200 μM). Cell growth rate in each group was determined on day 1, 3, 5, and 7 by Coulter counter as described in Methods. Results are shown as bar graphs of data obtained from three different experiments. **(C)** Wound healing assay for determining the migration capacity of the cells was carried out by making a cross-like scratch on the plate when they reached 90% confluency. Control and SAM (100 and 200 μM) treated cells were grown in culture media containing 2% FBS and migrating cells were photographed and recorded at different time points, and percentage of wound healing with respect to initial scratch (T0) was calculated using the equation described in ‘Supplementary Materials’. The results are represented as bar graphs obtained from three experiments. **(D)** Boyden chamber Matrigel invasion assay was used to measure the invasiveness of control and SAM-treated (100 and 200 μM) MDA-MB-231 and Hs578T cells. The cells were placed in the upper chamber, and conditioned media used as ‘chemoattractant’ was added into the lower chamber. Following an incubation period of 18 hours, the invasion process was stopped and the invaded cells from control and 100 and 200 μM SAM-treated groups were fixed, stained and randomly selected fields were counted under the microscope and averaged. Representative image of one randomly selected field for each treatment for both cell lines along with the number of cells invaded per field are shown. **(E)** After the usual treatment regimen, 5 × 10^3^ cell from control and SAM-treated (100 μM and 200 μM) groups were plated onto soft agar for anchorage-independent growth assay. The culture media was replenished every other day for two weeks, and the number of colonies was counted. **(F)** Apoptosis was determined by flow cytometry after staining the control and SAM-treated cells with Annexin V/propidium iodide. Representative contour plots of annexinV-FITC staining of apoptotic cells vs. PI staining for both control and SAM-treated (100 μM) cells are shown. The bar graphs on the right panels show the total percentages of apoptotic cells for different treatments. Results are presented as the mean ± SEM from control and SAM-treated experimental cells. Significant differences were determined using ANOVA followed by *post hoc* Bonferroni test and are represented by asterisks (^*^P < 0.05; ^**^P < 0.01, and ^***^P < 0.001).

Uncontrolled expansion of tumor cells through deregulated cell proliferation marks one of the critical events underlying the complexity and idiopathy of cancer cells [26]. Targeting cell proliferation has been one of the main focuses in cancer therapeutics. We, therefore, first examined the effect of SAM on the growth characteristics of two highly invasive human breast cancer cell lines MDA-MB-231 and Hs578T using our well-established experimental protocol (Figure 1A). Our results showed that treatment with two doses of SAM (100 μM and 200 μM) caused a significant dose-dependent reduction in tumor cell proliferation compared to vehicle-treated control cells, which demonstrates the anti-proliferative effect of SAM on breast cancer cells (Figure 1B). To determine whether SAM-treatment causes any adverse effect on the viability of normal non-tumorigenic cells *in vitro*, we treated normal human breast epithelial cells (HBEC) with the highest dose of SAM (200 μM) used in this study. Results from these studies showed that SAM-treatment did not cause any significant change in the percentage of viability in the treated cells compared to the control cells (Supplementary Table 2, Supplementary Figure 1). To determine the effect of SAM on cell migration, *in vitro* wound-healing capacity of control and SAM-treated (100 and 200 μM) MDA-MB-231 and Hs578T cells were assessed over a period of 48 hours from the initial scratch on the culture plate. The area of the initial scratch was similar for all the experimental groups. However, with the passage of time, control and SAM-treated cells displayed different migratory profiles during wound healing in both the cell lines. SAM treatment caused a significant dose-dependent decrease in the migratory ability of both breast cancer cell lines as compared to vehicle-treated control cells; effects which were most pronounced at 48 hours after the initial scratch (Figure 1C).

We next investigated whether SAM could suppress the invasiveness of MDA-MB-231 and Hs578T cells using Boyden chamber Matrigel invasion assay. Our *in vitro* data suggested that SAM-treatment caused a significant dose-dependent decrease in tumor cell invasion of both cell lines (Figure 1D).

We also evaluated the effect of SAM on anchorage-independent growth which is a hallmark of carcinogenesis *in vitro*. The ability of tumor cells to form colonies in soft agar allows for semi-quantitative evaluation of cellular transformation under different experimental conditions [27]. We observed a significant dose-dependent reduction of anchorage-independent growth by comparing the number of colonies formed by the control and SAM-treated (100 μM and 200 μM) cells from both cell lines (Figure 1E).

Next, to determine the effect of SAM on programmed cell death, an annexin V/PI apoptosis assay was performed using flow cytometry. As shown in Figure 1F, treatment with 200 μM of SAM caused a significant increase in the percentage of apoptotic cells in both cell lines as compared to the controls. To elucidate the potential mechanism of apoptosis, we determined the expression of anti-apoptotic Bcl-2 protein in control and experimental cells using Western blot analysis. These results a significant reduction in the expression of Bcl-2 in MDA-MB-231 cells treated with SAM as compared to the control cells (Supplementary File 1, Supplementary Figure 2). Results from these studies are consistent with the hypothesis that SAM mediates its apoptotic effects via suppressing anti apoptotic pathways such as the Bcl-2 signaling pathway. These results are in agreement with similar effects of SAM on other cancer cell types [28].

### SAM-treatment reduces tumorigenesis and metastasis in MDA-MB-231 xenograft mouse model

**Figure 2:**
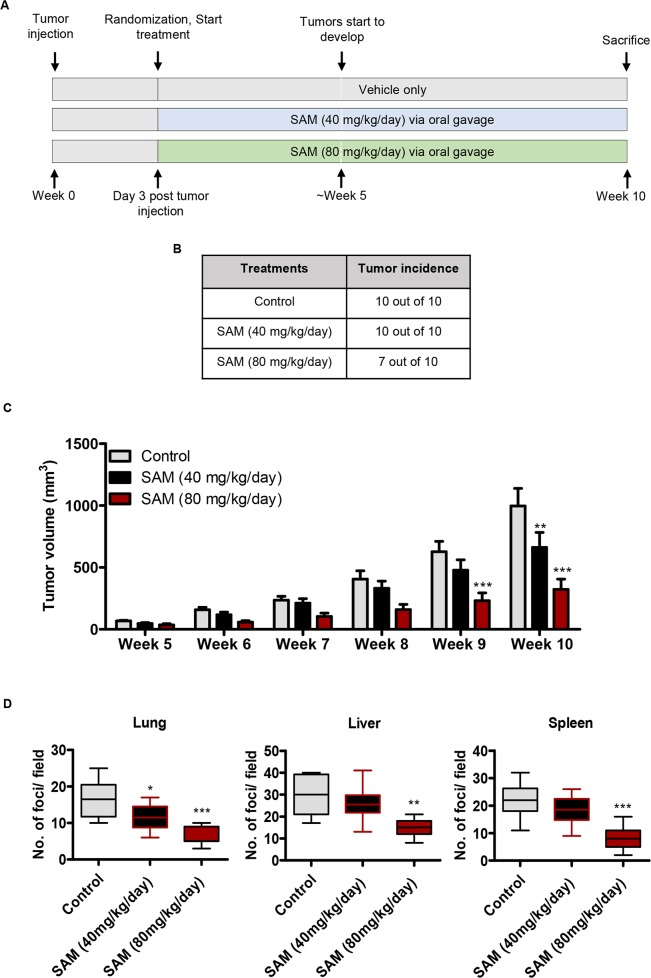
Effect of SAM on MDA-MB-231 tumor growth and metastasis **(A)** Schematic representation of SAM-treatment in MDA-MB-231 tumor xenograft mice. Female CD1 mice inoculated with MDA-MD-231-GFP cells via orthotopic route were randomized, and treatment with SAM at different doses was started from day three post tumor cell inoculation. Animals were treated daily with vehicle alone or SAM (40 mg/kg/day or 80 mg/kg/day) via daily oral gavage. **(B)** Tabular representation of the incidence of tumor in control and two experimental groups. **(C)** Tumor volume was determined at weekly intervals from week 5 when the animals started to develop tumors. Treatment with SAM caused a significant dose-dependent decrease in tumor growth. Results are representative of mean ± SEM of tumor volumes obtained from at least seven animals per group. Significant differences were determined using ANOVA followed by *post hoc* Bonferroni test and are represented by asterisks. (^**^P < 0.01; ^***^P < 0.001). **(D)** To evaluate the effect of SAM on tumor metastasis, control and SAM (40 and 80 mg/kg/day) treated animals were sacrificed at week 10 and different organs (lung, liver, spleen) were collected. Organ slices of 1-mm thickness were mounted on a glass slide, and the GFP-positive foci were examined under the fluorescent microscope. Ten randomly selected slides were counted and averaged to determine the GFP-positive metastatic foci in each organ. Significant differences were determined using ANOVA followed by *post hoc* Bonferroni test and are represented by asterisks. (^*^P < 0.05; ^**^P < 0.01, and ^***^P < 0.001).

Next, we moved to the principal aim of this study i.e. to assess the therapeutic potential of SAM in a xenograft model of breast cancer. MDA-MB-231-GFP cells were inoculated into the fat pad of the fourth mammary gland of immunodeficient female CD-1 nude mice, and the animals were treated with either vehicle only or two different doses (40 and 80 mg/kg/day) of SAM via daily oral gavage. A schematic representation of the treatment strategy is shown in Figure 2A. All the animals from vehicle-treated control, as well as the group receiving lower dose of SAM (40 mg/kg/day), developed primary tumors starting from week 5 which continued to grow until the sacrifice of animals at week 10 post tumor cell inoculation. In contrast, 3 out of 10 animals treated with higher dose of SAM (80 mg/kg/day) did not grow any primary tumor during the ten weeks of this study (Figure 2B). The treatment regimen using two different doses of SAM (40 and 80 mg/kg/day) via daily oral gavage showed a significant dose-dependent reduction in tumor volume as compared to the vehicle-treated control group (Figure 2C, Supplementary File 1, Supplementary Figure 3). SAM-treatment also showed a significant reduction in the weight of extirpated tumor compared to the controls after the sacrifice of all animals at week 10 (Supplementary File 1, Supplementary Figure 4). We did not observe any significant difference in the overall body weight of control and SAM-treated animals throughout the study (Supplementary File 1, Supplementary Figure 5).

We then assessed the anti-metastatic potential of SAM treatment. Lung, liver, and spleen of control and experimental animals were collected after sacrifice, and the number GFP-positive metastatic foci were counted. Experimental animals treated with 80mg/kg/day of SAM via daily oral gavage showed a significant reduction in the number of GFP-positive metastatic foci in lung, liver, and spleen as compared to vehicle-only controls (Figure 2D). However, the treatment with low dose of SAM (40mg/kg/day) didn't show anti-metastatic properties in all the organs (Figure 2D). Hence, further analysis was performed on the high dose (80 mg/kg/day) of SAM receiving group.

### SAM-treatment differentially regulates genes implicated in cancer progression and metastasis

**Figure 3:**
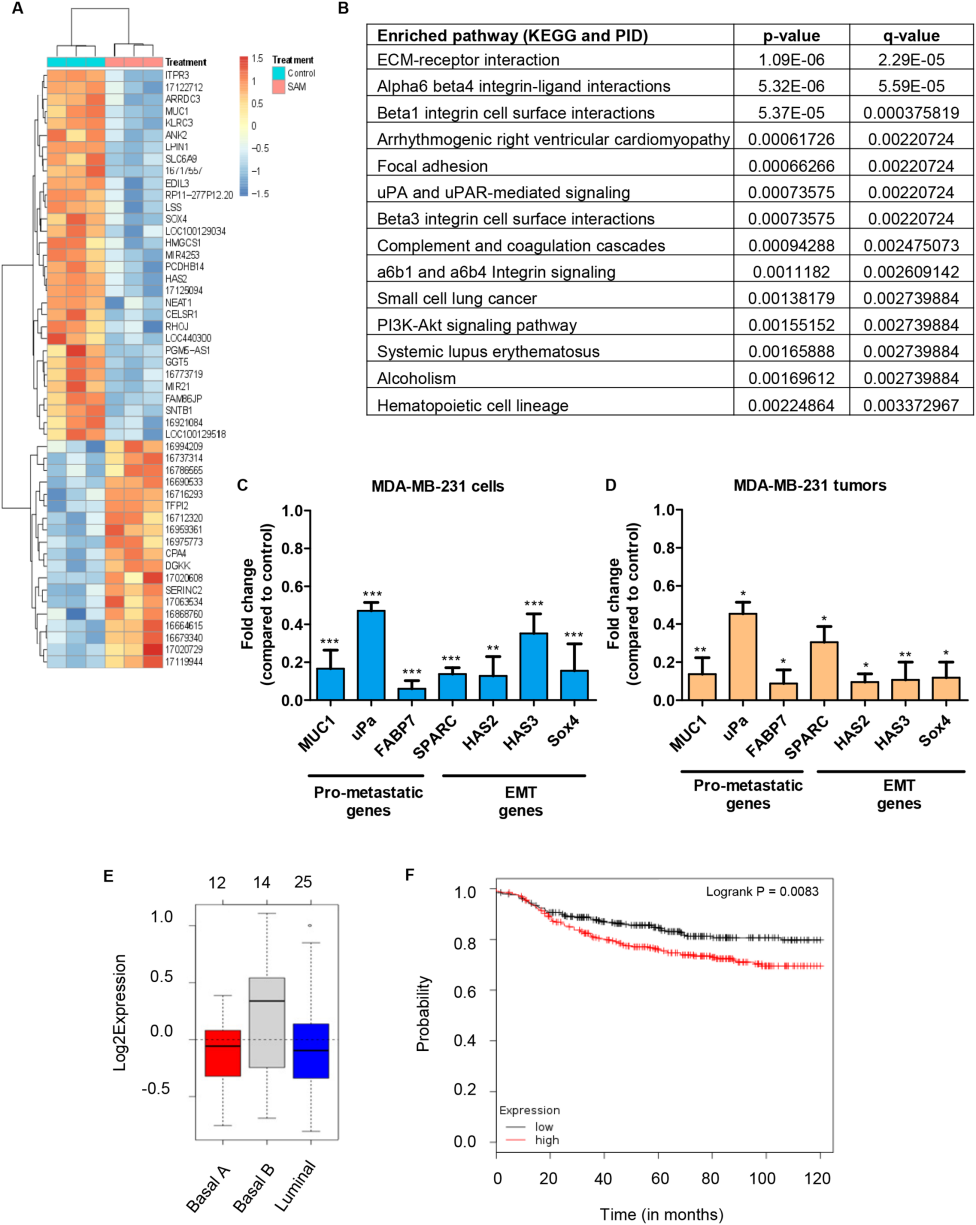
Gene expression analysis of MDA-MB-231 cells and tumors treated with SAM **(A)** MDA-MB-231 cells from control and SAM-treated (200 μM) group were subjected to Affymetrix array and the heat map of the most differentially expressed genes are shown (n=3 in each group). **(B)** Pathway analysis (from KEGG and PID database) of the genes that are differentially expressed upon SAM-treatment. **(C)** Selected genes differentially regulated by SAM were validated by quantitative real-time PCR (qPCR) in MDA-MB-231 cells. Results are shown as mean ± SEM of at least three independent experiments. (^**^P < 0.01, and ^***^P < 0.001). **(D)** RNA obtained from the tumor of control and 80 mg/kg/day SAM-treated animals were subjected to qPCR for the same set of genes that showed downregulation by SAM *in vitro*. Results are shown as mean ± SEM of at least three independent animals per group. (^*^P < 0.05 and ^**^ *P* < 0.01). **(E)** Gene Set Analysis (GSA) representing the expression of these genes in human breast cancer cell lines. **(F)** Kaplan-Meier plot of distant metastasis free survival from a dataset of 664 breast cancer patients categorized according to the expression of the seven down-regulated genes in Figure 3D.

We first evaluated the transcriptomic changes of MDA-MB-231 cells upon SAM-treatment. For that, we carried out microarray-based gene expression profiling (Affymetrix Human Gene 2.0 ST Array) using three independent sets of control and 200 μM SAM-treated RNA samples. We found that 476 microarray mRNAs were significantly altered in SAM-treated samples compared to controls (|fold change|>1.5 and *P*<0.01). A total of 231 microarray mRNAs were upregulated and 245 microarray mRNAs were downregulated in the SAM-treated samples when compared with control (Supplementary Table 2). Hierarchical clustering of top 50 most significantly changed microarray mRNAs are shown in Figure 3A.

Next, we analyzed the signaling pathways that were significantly altered upon SAM-treatment. The enriched pathway analysis of differentially regulated genes in breast cancer was performed using the Kyoto Encyclopedia of Genes and Genomes (KEGG) and Pathway Interaction Database (PID) databases. Our analysis showed that 14 pathways were significantly changed upon SAM-treatment (Figure 3B). Interestingly, most of the pathways that were altered by SAM-treatment have strong implication in cancer progression and metastasis.

To gain further insight into the biological processes affected by the genes that are differentially expressed upon SAM-treatment, we used WebGestalt [29] (Supplementary File 1, Supplementary Figure 6). Our analysis showed that the top biological process identified to be overrepresented by the genes upregulated by SAM-treatment functions in the negative regulation of endopeptidases (*P*=2.0 × 10^−7^; FDR=1.59 × 10^−3^). In contrary, the top hit for the genes downregulated by SAM is associated with positive regulation of cell-substrate adhesion (*P*=2.5 × 10^−6^; FDR=3.2 × 10^−2^). This further implies that SAM, through some unknown but surprisingly explicit mechanisms, plays a crucial role in regulating genes involved in tumor progression and metastasis.

Next, some of the genes identified through the expression array (*HAS2, Sox4, MUC1*) along with selected genes (*PLAU, SPARC, FABP7, HAS3*) implicated in cancer progression and metastasis were subjected to quantitative polymerase chain reaction (qPCR) analysis using the total RNA from control and 200 μM SAM-treated MDA-MB-231 cells. In experimental cells treated with SAM, a marked decrease in the expression of these genes was observed compared to vehicle-treated control cells (Figure 3C).

Next, RNA of primary tumors from control and experimental animals treated with 80 mg/kg/day of SAM were subjected to qPCR analysis. Similar to the results seen in the MDA-MB-231 cells *in vitro*, SAM-treatment *in vivo* reduced the expression of the 7 genes that were measured by qPCR analysis (Figure 3D). Gene set analysis revealed that in human breast cancer cell lines the expression of these seven genes (*MUC1, PLAU, FABP7, SPARC, HAS2, HAS3, SOX4*) are higher in basal-B subtype compared to other subtypes (Figure 3E). More interestingly, Kaplan-Meier analysis found significantly positive correlation between the higher expression of these seven genes and poor distant-metastasis free survival in breast cancer patients (Figure 3F). Collectively these results and data analysis shows that SAM can downregulate genes that have prognostic value for breast cancer metastasis.

### SAM-treatment changes promoter methylation status and protein expression of prometastatic genes

**Figure 4:**
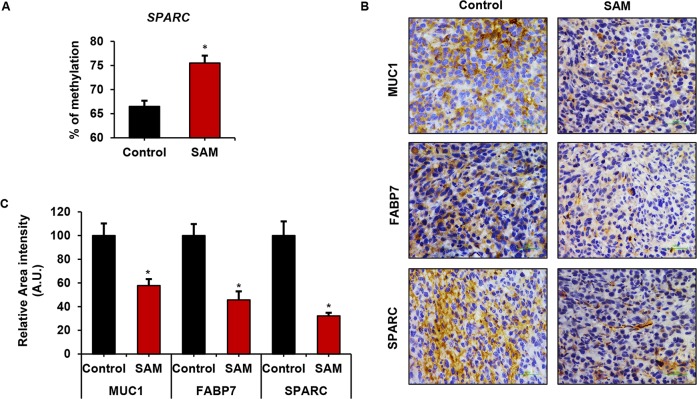
Effect of SAM-treatment on promoter methylation and protein expression of cancer-related genes **(A)** Site-specific methylation analysis by pyrosequencing at the promoter of *SPARC* (location: chromosome 5: 151066730; corresponding to Illumina 450K ID: cg22116670). **(B)** Immunohistochemistry of control and SAM-treated tumors using antibodies against MUC1, FABP7 and SPARC proteins. (C) The stained areas were quantified using Fiji plugin (ImageJ). Results are shown as mean ± SEM (n=3). ^*^P < 0.05.

We then focused on the methylation of promoters of prometastatic genes that were down-regulated by SAM treatment in the qPCR assay. Tumor DNA from experimental animals treated with SAM showed increased methylation of *SPARC* by pyrosequencing as compared to vehicle-treated control tumors (Figure 4A). We have previously shown the SAM-mediated methylation changes at the promoter of *PLAU* in breast cancer [23]. We didn't observe any significant methylation changes in the other genes (*MUC1, FABP7, HAS2, HAS3, SOX4*) that showed downregulation in qPCR (data not shown). There might be several possibilities behind such observations. First, the differentially methylated sites in response to SAM-treatment might be located beyond the regions that were focused on during pyrosequencing. Second, these genes are downstream of some other genes that are regulated by SAM, and the changes seen in qPCR are caused by indirect methylation effect of SAM on upstream genes. Third, SAM regulates these genes by a mechanism that is independent of DNA methylation such as histone methylation or other non-epigenetic mechanisms.

Next, we wanted to confirm the changes in protein expression in MDA-MB-231 tumors in response to SAM-treatment by immunohistochemical analysis. As shown by the representative image of control and SAM-treated tumors probed with antibodies for MUC1, SPARC, and FABP7 in Figure 4B-4C, a significantly reduced staining of these proteins were observed in SAM-treated tumors compared to the control tumors. The SAM-mediated changes in protein levels of PLAU have been previously shown by our group [6, 23]. We were unable to determine the change in the expression of SOX4, HAS2, and HAS3 proteins due to lack of well-characterized antibodies with a higher specificity of staining pattern.

### SAM is bioavailable in the serum of experimental animal with no adverse behavioral and physiological changes

**Figure 5:**
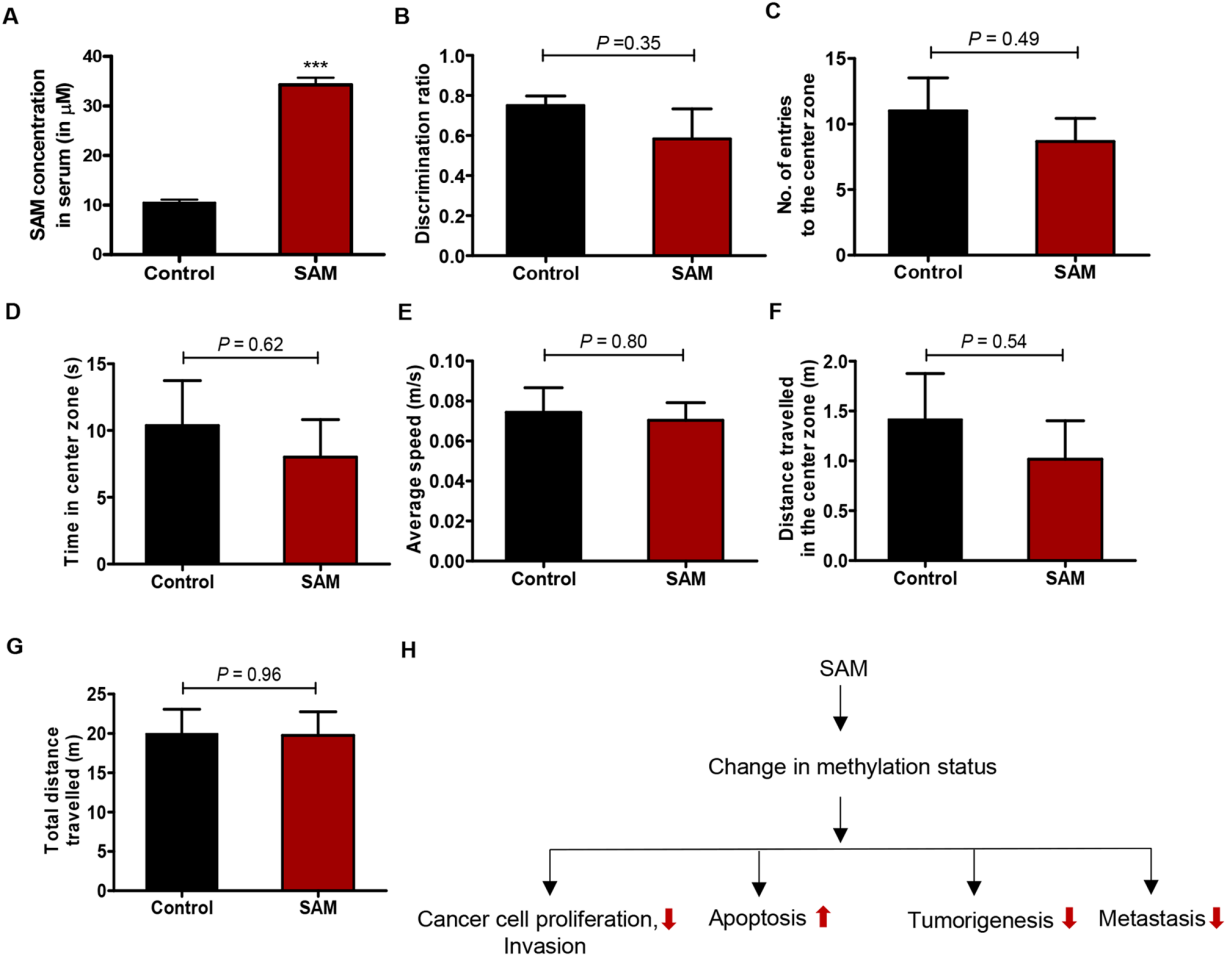
Assessment of bioavailability and animal behavior upon SAM-treatment **(A)** The average level of SAM in control and the experimental group receiving 80 mg/kg/day ofSAM as determined by the SAM ELISA. Results are obtained from the analysis of serum from four animals in each group. (^***^P < 0.001). **(B)** Novel object recognition test of control and SAM-treated mice. Average Discrimination ratio (time spent with the novel object/ total time spent with both object). No significant differences in cognitive abilities are detected between control and SAM-treated groups. **(C-G)** Different parameters determined by the open field test of control and SAM-treated mice also showed no significant difference between control and SAM-treated animals. Results are shown as mean ± SEM (n=3 for each group of CD-1 nude mice), and statistical analyses were done using student's *t*-test. **(H)** A summary of biological processes shown to be affected by SAM as determined in this study includes cell proliferation, invasion, apoptosis *in vitro* and tumorigenesis and metastasis *in vivo*.

Lack of bioavailability often hinders the therapeutic potential of anti-cancer agents. To be efficacious, the therapeutic molecule needs to be available in the blood for a reasonable amount of time so that it can be absorbed and then circulated to the target organ(s). Towards these goals, serum from control and experimental animals were analyzed for the presence of SAM using an enzyme-linked immunosorbent assay (ELISA). We found that the average basal level of SAM in the control animals was 10.43 ± 0.57 μM which increased to 34.22 ± 1.45 μM in the treatment group receiving 80 mg/kg/day of SAM (Figure 5A). We also performed a relative analysis of the SAM levels in the serum of control and experimental animals treated with exogenous SAM by LC-MS/MS and observed a similar increase in the levels of SAM in experimental group of animals (data not shown). This confirms that SAM is bioavailable in the animals after administration through oral gavage suggesting that it might also be orally available in humans.

Even though SAM is widely used as a supplement for depression, some transient adverse behavioral effects were previously reported in human clinical trials [30]. To assess whether SAM treatment causes any behavioral change at the efficacious dose of this study i.e. 80 mg/kg/day, we next conducted two different behavioral tests on control and SAM-treated mice. First, a novel object recognition test measuring the cognitive function of mice was performed. We didn't observe any difference between the control and experimental group of animals in the quest for exploration for the novel object (Figure 5B).

Next, we performed the open field test. This test is used to evaluate any potential anxiolytic or anxiogenic effect of a therapeutic agent by measuring locomotion related anxiety levels of experimental animals placed inside an open field box. The open field test is based on the concept that the natural instinct of mice is to stay in proximity to the protective wall rather than exposing themselves to danger in the open areas [31]. When control and SAM-treated mice were exposed to an open field apparatus, there was no significant difference in the frequency and time spent in the central region (Figure 5C-5D). Moreover, both control and SAM-treated mice moved around at almost similar speed (Figure 5E), and there was also no significant difference in the total distance traveled within the central zone as well as the whole experimental arena (Figure 5F-5G). Taken together, these observations suggest that SAM does not cause any detrimental behavioral defects at the doses used in this study.

When serum from control and experimental animals were analyzed for different biochemical measurements (liver function test, kidney function test, major electrolytes/minerals), we didn't see any significant changes in the SAM-treated animals compared to control animals (Supplementary File 1, Supplementary Table 1). This suggests that SAM is non-toxic at the highest dose (80 mg/kg/day) used in this study.

In summary, we have shown that SAM-treatment reduced proliferation, invasiveness of breast cancer cells and increased apoptosis *in vitro*, and reduced tumorigenesis and metastasis *in vivo* (Figure 5H).

## DISCUSSION

A large body of evidence has shown that abnormal DNA methylation is associated with cancer development and progression [32, 33]. Both hypomethylation and hypermethylation are involved in cancer [34]. Despite that, the focus of attention for the past two decades has been on targeting hypermethylation by the administration of inhibitors of DNA methyltransferase enzyme (DNMTi). Two inhibitors, 5-Azacytidine (Vidaza®) and 5-aza-2'-deoxycytidine (Dacogen®), have already received FDA-approval for the treatment of several specific forms of myelodysplastic syndromes (MDS), acute myeloid leukemia (AML), and chronic myelomonocytic leukemia (CMML) and additional clinical trials are ongoing for several other cancers [20, 35]. However, the activity of DNMTi has been limited in the case of the solid tumors largely due to toxicity and lower stability of these drugs [36, 37]. In addition, these drugs also promote the invasiveness in cancer cells through hypomethylation-mediated upregulation of prometastatic genes [16].

Accumulating evidence support the fact that there are broad regions of hypomethylation in the cancer genome and that hypomethylation is prevalent at promoters [38, 39]. Epigenome-wide association studies (EWAS) in osteosarcoma, prostate, and liver cancer revealed that the promoters of a large number of genes involved in tumor growth and metastasis are hypomethylated [21, 22, 39]. These findings lead to the hypothesis that agents that induce hypermethylation at the promoters of metastatic genes would repress tumor metastasis.

Although the exact reason behind the increase in hypomethylation during the progression of the disease is still an enigma in the field of cancer epigenetics, Hoffmann and Schulz suggested that this might be partly due to inadequate amounts of the methyl group donor SAM [40]. Treatment with SAM has been shown to trigger hypermethylation of several genes in cell culture experiments [23]. To date, SAM is the only therapeutic agent that is known to cause hypermethylation of DNA and silencing of hypomethylated genes in cells. SAM is attractive as a therapeutic agent since it is an approved natural supplement and has a very good safety profile.

Although past studies provided evidence that SAM has antiproliferative and anti-metastatic effects *in vitro* against breast cancer cells and this was replicated in this study using two different basal-like breast cancer cell lines (MDA-MB-231 and Hs578T), the critical question that remained to be answered was whether SAM was effective as an oral therapeutic agent under conditions that could be replicated in breast cancer patients. In the current study, we tested whether *in vivo* supplementation of SAM would exhibit anti-proliferative and anti-metastatic effects in a xenograft model of breast cancer *in vivo*. Our study demonstrated that oral administration of SAM caused a significant dose-dependent reduction in mammary tumor volume and metastasis in our well-characterized xenograft model of breast cancer, holding great promise for translating similar treatment strategies to breast cancer patients. It should be noted that our results demonstrate responses in basal-like breast cancer cells (MDA-MB-231) which are highly aggressive, and patients with such type of breast cancer have shorter survival rate compared to other types of breast cancer patients [41]. In addition, unlike other subtypes, there is still no known target for basal-like breast cancers which warrants continued efforts to develop effective therapeutic approaches for this group of patients. We hypothesize that if SAM can show favorable outcome in the most aggressive form of breast cancer, it can be more easily translated into other subtypes as well. Since SAM is an accepted orally bioavailable nutritional supplement, it might be used in a preventative setting to prevent recurrence and metastasis post surgery.

Another aspect of the current study was to assess the underlying molecular changes pertaining to SAM-treatment both *in vitro* and *in vivo*. Towards achieving this goal, we first examined the changes in the expression of genes implicated in tumor metastasis by selecting a combination of genes already known to have a role in cancer along with those selected by a gene expression array on MDA-MB-231 cells. Our microarray-based transcriptome-wide analysis as well as qPCR validation showed that SAM-treatment caused downregulation of several genes implicated in cancer progression and metastasis (Figure 3C & 3D). More importantly, the gene expression changes observed in the cell lines could be recapitulated in the xenograft tumors. When qPCR was performed using the same set of genes that were downregulated *in vitro*, they showed similar downregulation in tumor RNA extracted from SAM-treated animals as compared to vehicle-treated controls. Such reduction in the expression of these genes might be either due to promoter methylation in response to SAM-treatment or an indirect effect in which SAM caused the methylation and silencing of critical activators or enhancers of transcription of these genes. Previously we have shown that SAM-treatment caused direct methylation in the promoter of *PLAU* [23]. In this study, we found a marked increase in methylation at the promoter of *SPARC* in the SAM-treated xenograft tumor DNA as compared to controls, suggesting promoter methylation effect of SAM on this promoter as well. However, we did not observe any significant change in methylation in other genes (*MUC1, FABP7, SOX4, HAS2, HAS3*) that were tested through pyrosequencing. These genes might be regulated indirectly by DNA methylation of other genes which are required for their activation. Alternatively, SAM might suppress these genes by other epigenetic mechanisms such as histone methylation or non-epigenetic mechanisms. Further experiments are required to address this question. We also validated the SAM-mediated downregulation of three proteins (MUC1, SPARC, FABP7) by immunohistochemistry.

To confirm the bioavailability of SAM, we performed an ELISA-based assay and found a significant increase in the level of SAM in experimental animals compared to non-treated controls. SAM was bioavailable at the dose used in this study and caused changes in the expression levels of genes present in the mammary tissue to reduce or inhibit cancer cell growth and metastasis.

A major concern with the use of hypermethylating agent is the possible silencing of tumor suppressor genes through hypermethylation of promoter and other regulatory regions. Such methylation could override the beneficial effect of SAM. When we checked the expression of some of recognized tumor-suppressor genes in MDA-MB-231 tumors, there was no significant difference between control and SAM-treated groups (Supplementary File 1, Supplementary Figure 7). This also complements our previous genome-wide analyses in prostate cancer and osteosarcoma cell lines where the methylation effect of SAM was limited to cancer-promoting genes for yet unknown reasons [21, 22]. More interestingly, database search using the panel of seven genes (*MUC1, PLAU, FABP7, SPARC, HAS2, HAS3, SOX4*) downregulated by SAM revealed that these genes are highly expressed in basal B-type breast cancer cell lines and higher expression of these genes significantly decreases the probability of distant metastasis-free survival in breast cancer patients [42].

It has been previously suggested that SAM shows selective cytotoxicity for cancer cells and not for normal cells [43]. SAM-treatment did not have any significant effect on the viability of normal human breast epithelial cells at the highest dose used in this study (Supplementary File 1, Supplementary Figure 1). This further verifies that SAM is not cytotoxic to the normal breast epithelial cells. We also performed extensive biochemical analysis of the blood samples collected from SAM-treated animals and observed no significant changes in any of the parameters tested as compared to controls (Supplementary File 1, Supplementary Table 1). In addition, our study demonstrated that SAM-treatment also didn't cause any adverse behavioral changes as shown by novel object test and open field test.

The main question that pertains to numerous other pharmacological agents as well, is how does a general methylating agent such as SAM target only a subset of genes and has an effective anticancer effect with very little adverse effect on normal tissue. We have recently investigated this question at the genomic level in normal and liver cancer cell lines by analyzing the transcriptome and methylome of normal and cancerous cells treated with SAM (Wang et al., Oncotarget, in press). It appears that the matrix of the transcriptome and methylome that SAM acts upon in normal and cancer cells is very different and that the outcome of this interaction between a general agent and an exquisite transcription and methylation landscape appears to be different. SAM does not methylate DNA on its own, DNMTs do. The consequence of an elevation in SAM levels is dependent on the pre-existing distribution of DNMTs. Similarly, inspection of the vast literature on DNA methylation inhibitor 5azaC shows that demethylation results in different transcription and cell fate consequences, for example myogenesis and induction of muscle-specific genes in fibroblasts and globin genes in erythroleukemia cells. The most plausible explanation is that modulation of DNMT activity is restricted by the distribution of DNMTs and factors that regulate the accessibility of DNMTs across the genome and these define the specific outcome of modulation of DNMTs by either methyl donors or DNMT inhibitors.

This study examined the involvement of DNA methylation in mediating SAM cellular effects and provided evidence for silencing of several prometastatic genes as a plausible mechanism for SAM action on metastatic breast cancer. But it is most probable that the alteration of DNA methylation is just one of several mechanisms through which SAM exerts its effects. SAM is a pleiotropic molecule, and acts as a methyl group donor to other biological substrates like RNAs, proteins, lipids and small molecules [44]. Therefore, it is likely that SAM exerts its anti-cancer effect through biochemical pathways in addition to DNA methylation. It is possible that SAM-treatment alters the methylation status of histone proteins which in turn interfere with the chromatin architecture to make the promoters of the cancer-promoting genes inaccessible for transcription factor binding. The pleiotropic effect is evident by the changes seen in multiple cellular processes like tumor cell proliferation, invasion, and apoptosis upon SAM-treatment. Further detailed studies are required to explore these mechanisms to extend our understanding of how SAM exerts these effects.

To our knowledge, this is the first direct evidence for the potential therapeutic effect of SAM in a well-recognized model of breast cancer. Results from these studies provide compelling evidence to evaluate the therapeutic as well as a chemopreventive potential of epigenetic-based agents such as SAM alone and in the combination setting for patients with several common cancers including breast cancer.

## MATERIALS AND METHODS

### Cell culture and treatments

The cell lines were obtained from the American Type Culture Collection (ATCC; Manassas, Virginia). The MDA-MB-231(*ATCC*® HTB- 26™) human breast cancer cells were maintained in Dulbecco's modified Eagle's medium (DMEM) supplemented with 10% fetal bovine serum (FBS), 2 mM L-glutamine and 100 units/ml penicillin-streptomycin sulfate at 37°C and 5% CO_2_. For Hs578T (*ATCC® HTB-126™*) cells, DMEM containing 10% FBS, 1.25 mg/mL insulin, 2 mM L-glutamine and 100 units/ml penicillin-streptomycin sulfate was used. These cell lines were routinely examined on their viability, cellular morphology, growth patterns and microbial presence by microscopic observation. The cell lines were authenticated by the Genetic Analysis Facility, The Hospital for Sick Children, Toronto. The human breast epithelial cells (HBEC) were purchased from Celprogen (Cat# 36056-01) and were maintained in commercially available human breast epithelial cell culture serum free media (Celprogen, Cat# M36056-01).

Cells were treated with SAM (New England Biolabs, Mississauga, Ontario, Canada; Catalog # B9003S) by directly adding it to regular growth medium under sterile conditions following the treatment plan shown in Figure 1A. Different doses of SAM ranging between 25–500 μM were previously tested by our group for *in vitro* efficacy in different cancer cell lines [5, 6, 21, 22]. In this study, the effect of 100 and 200 μM doses of SAM were evaluated.

### Cell proliferation, migration, invasion and anchorage-independent growth assay

These assays were done according to our previous studies [21, 22]. Details are available in the ‘Supplementary Materials’.

### Apoptosis assay

For apoptotic assays, 1×10^6^ cells from control and SAM-treated groups were stained using ‘Dead Cell Apoptosis Kit’ (TheremoFisher, Cat# V13242, Eugene, Oregon, USA) according to the manufacturer's instructions. The apoptotic cells were detected using recombinant annexin V conjugated to green fluorescent FITC dye, and dead cells were detected using propidium iodide (PI). Stained cells were then analyzed using a BD FACSCanto II flow cytometer (BD Biosciences, San Jose, California, USA). For data acquisition and analysis of apoptotic events, BD FACSDiva^™^ (BD Biosciences) and FlowJo software (FlowJo LLC, Ashland, OR, USA) were used, respectively.

### Study approval and *in vivo* xenograft model

All the *in vivo* procedures carried out during this study were done in compliance with a protocol approved by the McGill University Facility Animal Care Committee. Female CD-1® Nude mice aged between 4-6 weeks were obtained from Charles River, St-Constant, Quebec, Canada and maintained at the Animal Resource Division of the McGill University Health Center. This is a well-established mouse model used for the studies related to tumor xenografts [45–47]. Highly invasive MDA-MB-231 cells expressing green fluorescent protein (MDA-MB-231-GFP), which have the capacity to metastasize to different secondary organs [6], were used for inoculation into the immunodeficient mice. Briefly, mice were inoculated with 5×10^5^ MDA-MB-231-GFP cells with 20% Matrigel (BD Biosciences) into the fat pad of the fourth mammary gland. Three days post-inoculation; the animals were randomized into three different groups: phosphate buffer saline (PBS) as the vehicle-treated controls, a group receiving 40 mg/kg/day of SAM and another group receiving 80 mg/kg/day of SAM via oral gavage. We have used SAM from two sources (New England Biolabs, Mississauga, Ontario, Canada and Life Science Laboratories, Lakewood, NJ, USA) which showed similar anti-cancer effects in our *in vitro* studies (data not shown). However, since SAM from Life Science Laboratories is human-grade, it was used for all *in vivo* studies since this product could be also used in future clinical trials in patients with breast cancer.

Tumor diameters were determined weekly using a Vernier caliper for a 10-week period after inoculation, and tumor volume was calculated using the following formula: V= (length × Width^2^)/2. At the end of the study period, the animals were sacrificed and different tissues were collected for further analysis.

For studying metastasis, the harvested lung, liver and spleen were cut into 1-mm thick slices, smeared on a glass slide, and placed under a fluorescent microscope for detecting the presence of GFP-expressing tumor foci. Randomly selected fields were counted for the presence of GFP-positive foci in each organ, and the average number of foci per group was graphed.

### RNA extraction and quantitative real-time PCR (qPCR)

Total RNA from the cell lines and xenograft tumors was extracted using the RNeasy kit (Qiagen; Hilden, Germany, Cat# 71404) and AllPrep DNA/RNA Mini Kit (Qiagen; Cat# 80204) respectively following the manufacturer's protocol. The qPCR assay was performed following our previously described protocol [22]. The primers are listed in Supplementary Methods Table 1. Gene expression changes between control and SAM-treated samples were carried out using the 2^-ΔΔC^T method.

### Gene expression microarrays

For gene expression array, 100 nanograms of total RNA from control and 200 μM SAM-treated MDA-MD-231 samples from three independent experiments was used. RNA quality and quantity were assessed using NanoDrop® ND-1000 spectrophotometer (Thermo Scientific, Wilmington, Delaware, USA) (260/280 >1.8 accepted) and Agilent 2100 Bioanalyzer (Waldbronn, Germany) (RIN 7 ≥ accepted). Gene expression profiling was performed using Affymetrix Human Gene 2.0 ST Array (Santa Clara, California, USA) at the Génome Québec Innovation Centre (McGill University) following standard protocols.

Data from the biological replicates were then normalized using the Robust Multi-array Average (RMA) method implemented in the Bioconductor package *oligo* [48]. Differential gene expression analysis was performed using the Bioconductor package Limma with a threshold defined by *P* <0.01 and |fold change| >1.5. The data was submitted to Gene Expression Omnibus (GEO) under the accession number of GSE98275.

### Determination of SAM levels in the serum by ELISA

To assess bioavailability, ELISA was done using serum from experimental mice collected within 1-hour post oral administration of SAM. Serum from control mice was also obtained for comparison. Then ELISA (myBioSource, San Diego, CA, USA, Cat# MBS169240) was performed according to the manufacturer's protocol. The level of SAM was extrapolated from the curves obtained from the manufacturer provided synthetic standards of SAM.

### DNA extraction, Bisulfite conversion, and Pyrosequencing

Genomic DNA from the tumors was extracted using AllPrep DNA/RNA Mini Kit (Qiagen) and bisulfite conversion was conducted using the EZ DNA Methylation-Gold Kit (Zymo Research, Irvine, CA, USA; Cat#D5005). Selected regions from the bisulfite converted sequences were then amplified with Taq DNA polymerase (Thermo Fisher Scientific, Lithuania, EU; Cat# EP0402) using biotinylated primers (listed in Supplementary Materials, Supplementary Table 1). Then pyrosequencing was conducted on the biotinylated DNA strands using PyroMark Q24 instrument (Biotage, Qiagen). For post-run data analysis, PyroMark Q24 software (Qiagen) was used.

### Western blot and immunohistochemistry

Details are available in the ‘Supplementary Materials’.

### Behavior test

To assess any potential behavior adversities induced by SAM-treatment novel object recognition test and open field tests were done. Details are available in the ‘Supplementary Materials’.

### Statistical analysis

The results are expressed as mean ± standard error of the mean (SEM). Depending on the experimental design, statistically significant differences between different quantitative measurements were carried out by two-tailed Student's *t*-test, one-way or two-way ANOVA. ^*^P < 0.05, ^**^P < 0.01, and ^***^P < 0.001 were considered statistically significant. Gene set enrichment analysis (GSEA) was carried out by using ConsensusPathDB [49]. The association between the expression of the different cancer-related gene and distant metastasis-free survival was determined using Kmplotter [42]. Gene expression-based outcome analysis of breast cancer was carried out by GOBO [50].

## SUPPLEMENTARY MATERIALS FIGURES AND TABLES




