# Supplementary material for: Methyl donor S-adenosylmethionine (SAM) supplementation attenuates breast cancer growth, invasion, and metastasis in vivo; therapeutic and chemopreventive applications
Source: Oncotarget. 2017 Dec 26;9(4):5169–83. doi: 10.18632/oncotarget.23704 (PMC5797041; doi:10.18632/oncotarget.23704)
Supplement: Supplementary file 1 [file oncotarget-09-5169-s001.pdf]

Methyl donor S-adenosylmethionine (SAM) supplementation attenuates breast cancer growth, invasion, and metastasis *in vivo*; therapeutic and chemopreventive applications

SUPPLEMENTARY MATERIALS

Cell proliferation and viability assay

For proliferation assay, MDA-MB-231 and Hs578T cells plated in each well of 6-well plates were treated with 100 and 200 µM SAM or vehicle every second day for six days (Figure 1A). The cells were trypsinized and counted at different time points starting from day 1 (no treatment) until the end of each treatment period (on day 7 from the initial plating) using a Coulter counter (Model ZF; Coulter Electronics, Hertfordshire, UK). For viability assay, cells were trypsinized, stained with 0.4% trypan blue (Sigma) and the viable cells were counted under a light microscope.

Cell migration/ wound healing assay

For *in vitro* wound healing analysis, MDA-MB-231 and Hs578T cells were treated with 100 and 200 µM SAM or vehicle following the treatment strategy mentioned in Figure 1A in the presence of regular cell culture media supplemented with 10% FBS in 10 cm Petri dishes. Afterward, the cells were trypsinized, and 500,000 cells were plated in each well of 6-well plates to form a monolayer and then wounded manually with a sterile 200 µL pipette tip in the center of each well forming a cross-like section of the wound. Cells were then washed twice with serum-free culture medium to get rid of the detached cells and debris. From this point, the cells were grown in the presence of culture media supplemented with 2% FBS and migrating cells were photographed at different time points (0, 6, 24, 48 hours after initial wounding) with an inverted bright field microscope under the 4X objective. Analysis and quantification of the cell-free area were carried out using the Image Pro-Plus software (Media Cybernetics, Inc, Rockville, MD, USA). The measurements obtained from the software were calculated as percentage wound healing using the equation: % wound healing = [1 − (wound area at T_xh/wound area at T_0)], where T_x is the respective time point, and T_0 is the initial time immediately after the scratch.

Boyden chamber matrigel invasion assay

The changes in the invasive capacity of control and SAM-treated samples of MDA-MB-231 and Hs578T breast cancer lines was tested using a two-compartment Boyden chamber invasion assay (Costar Transwell, Corning Corporation, Sigma-Aldrich, Oakville, Ontario, Canada). The 8-µm-pore polycarbonate filters provided by the manufacturer were first coated with basement membrane Matrigel (50 µg/filter). Briefly, 2.5 × 10^5 viable cells from different treatment groups were resuspended in 100 µL of serum-free culture media and added to the upper chambers of the Matrigel Boyden wells. 800 µL of conditioned media was added to the lower chamber as the chemoattractant. After an incubation period of 18 hours at 37°C with 5% CO_2, the invasion assay was stopped by moving the cells out of the incubator. The upper chamber was then washed with PBS to remove the non-invading cells from the top of the membrane. Then invading cells at the bottom of the membrane were fixed using 2% paraformaldehyde and 0.5% glutaraldehyde (Sigma-Aldrich) in 0.1 M phosphate buffer saline (PBS) with a pH of 7.4, at room temperature for 30 minutes. The membranes were then stained with 1.5% toluidine blue, washed with PBS, mounted onto glass slides, and the number of invading cells from randomly selected fields were counted under a light microscope and averaged.

Colony formation assay

To determine the effect of SAM on anchorage-independent growth, a measure of cellular transformation *in vitro*, soft agar colony formation assay was performed as previously described [1]. Briefly, 5 × 10^3 MDA-MB-231 and HS578T cells from control SAM-treated groups were counted and seeded in triplicates onto 6-well Petri dishes (BD Falcon™) in the presence of 4 ml of complete culture medium containing 1.5% agar (Bioshop®; Catalog# AGR001) solution. The culture medium was replenished every second day and the colonies formed after 2 weeks were counted under a light microscope.

Western blot

Cell lysates from control and SAM-treated cells were prepared using radioimmunoprecipitation assay buffer (RIPA) containing a cocktail of protease and phosphatase inhibitors. Equal amounts of proteins were loaded and resolved on a 15% sodium dodecyl sulfate-polyacrylamide gel and then transferred to polyvinylidene difluoride (PVDF) membrane using standard protocols. After transfer, non-specific binding was blocked by using 5% milk in

Tris-buffered saline (TBS). Mouse monoclonal Bcl-2 antibody (Santa Cruz Biotechnology, Cat# sc-7382) was used to detect the anti-apoptotic Bcl-2 protein, and anti-mouse β-tubulin (BD Pharmingen cat# 556321) was used a loading control. The anti-mouse secondary antibodies used in this study were purchased from Bio-Rad, and the proteins were visualized by using an enhanced chemiluminescence detection kit (Amersham, GE Healthcare Life Sciences).

Immunohistochemistry

Immunohistochemistry was performed on paraffin-embedded sections, cleared with xylene. Heat-mediated antigen retrieval was carried out by Tris/EDTA pH 9.0 buffer, Envision™ FLEX Target Retrieval Solution (Dako, Denmark) at 1:50 dilution for SPARC, MUC1 and FABP7. Endogenous peroxidase activity was blocked by Envision FLEX Peroxidase-Blocking Reagent (Dako). Antibodies for SPARC, MUC1, and FABP7 (Abcam, Cambridge, UK) were used at 1:250, 1:100, and 1:200 dilutions respectively as primary antibodies. Horseradish Peroxidase (HRP)-conjugated secondary antibody was used. EnvisionTM FLEX DAB+ Chromogen (Dako) and EnvisionTM FLEX Substrate buffer (Dako) were applied. The slides were counterstained with hematoxylin (MERCK, NJ, USA). Sections were washed twice for 10 minutes in Tris buffered saline solution pH7.6 (EnvisionTM FLEX Wash Buffer, DAKO) at 1:20 dilution after every step during the procedure. Slides were mounted with DPX (MERCK). The stained areas from randomly selected fields were then quantified using ImageJ (Fiji plugin) (National Institutes of Health, USA).

Novel object recognition test

For the novel object recognition test to assess whether SAM-treatment has any adverse effect on memory, animals were allowed to explore two identical copies of the first object in an open field enclosure for 10 minutes. By this time, the first object is now familiar to the mouse. Then there was a 60-minute break before the assessment of short-term memory began. During this period, the animals explored a familiar object (first object used during training) for eight minutes and a novel object (different shape) for 5 minutes and the time spent in exploration of the objects were recorded. Discrimination index (DI), a ratio of the time spent with the novel object in comparison with the familiar object, was determined and used for comparing between control and SAM-treated animals [2].

Open field test

The open field test to assess any potential increase in the anxiety levels upon SAM-treatment was conducted by placing mice from control and treatment group in an open field arena measuring 45 × 45 × 60 cm. The mice were tested individually for 5 minutes, their movement activity during this period was recorded using a camera and later analyzed by ANY-maze software. During the analysis stage, the open field arena was partitioned into nine squares having similar areas using the ANY-maze software. The square in the center was regarded as the central zone, and the surrounded areas were called the peripheral zone. Different parameters like the frequency and time spent in the center, total distance traveled within the central zone as well as the whole open field box along with their locomotion speed were calculated by ANY-maze, and the results were shown in bar graphs.

REFERENCES

1. Shukeir N, Stefanska B, Parashar S, Chik F, Arakelian A, Szyf M, Rabbani SA. Pharmacological methyl group donors block skeletal metastasis *in vitro* and *in vivo*. Br J Pharmacol. 2015; 172:2769-81.
2. Ennaceur A, Delacour J. A new one-trial test for neurobiological studies of memory in rats. 1: behavioral data. Behav Brain Res. 1988; 31:47-59.
3. Cheishvili D, Chik F, Li CC, Bhattacharya B, Suderman M, Arakelian A, Hallett M, Rabbani SA, Szyf M. Synergistic effects of combined DNA methyltransferase inhibition and MBD2 depletion on breast cancer cells; MBD2 depletion blocks 5-aza-2';-deoxycytidine-triggered invasiveness. Carcinogenesis. 2014; 35:2436-46.
4. Tiwari N, Tiwari VK, Waldmeier L, Balwierz PJ, Arnold P, Pachkov M, Meyer-Schaller N, Schübeler D, van Nimwegen E, Christofori G. Sox4 is a master regulator of epithelial-mesenchymal transition by controlling Ezh2 expression and epigenetic reprogramming. Cancer Cell. 2013; 23:768-83.
5. Chaika NV, Gebregiworgis T, Lewallen ME, Purohit V, Radhakrishnan P, Liu X, Zhang B, Mehla K, Brown RB, Caffrey T. MUC1 mucin stabilizes and activates hypoxia-inducible factor 1 alpha to regulate metabolism in pancreatic cancer. Proc Natl Acad Sci U S A. 2012; 109:13787-92.
6. Eißmann M, Gutschner T, Hämmerle M, Günther S, Caudron-Herger M, Groß M, Schirmacher P, Rippe K, Braun T, Zörnig M. Loss of the abundant nuclear non-coding RNA MALAT1 is compatible with life and development. RNA Biol. 2012; 9:1076-87.
7. Liu J, Tu F, Yao W, Li X, Xie Z, Liu H, Li Q, Pan Z. Conserved miR-26b enhances ovarian granulosa cell apoptosis through HAS2-HA-CD44-Caspase-3 pathway by targeting HAS2. Sci Rep. 2016; 6:21197.

Supplementary Methods Table 1: Primers used in this study are listed below [Source: [3–7]]

Gene Name		Sequences used for qPCR (5'→ 3')
uPA	For	TTCGGAGGGCAGCACTGTGAAATA
	Rev	GCATGGTACGTTTGCTGAAGGACA
SPARC	For	TCACATTAGGCTGTTGGTTCAAA
	Rev	CGCTGACCACTTCCCAGAGA
FABP7	For	TGCTTGCTGAGGTGTAAAGGGTCT
	Rev	TGACTGTTGGTCAGCTTCCAGGTA
HAS3	For	GGTACCATCAGAAGTTCCTAGGCAGC
	Rev	GAGGAGAATGTTCCAGATGCG
SOX4	For	CCAAATCTTTTGGGGACTTTT
	Rev	CTGGCCCCTCAACTCCTC
MUC1	For	CTGCTCCTCACAGTGCTTACAGTTG
	Rev	TGAACCGGGGCTGTGGCTGG
NEAT1	For	CCAGTTTTCCGAGAACCAAA
	Rev	ATGCTGATCTGCTGCGTATG
HAS2	For	TTATGGGCAGCCAATGTA
	Rev	ACTTGCTCCAACGGGTCT
NEAT1	For	CCAGTTTTCCGAGAACCAAA
	Rev	ATGCTGATCTGCTGCGTATG
PTEN	For	TGTGCTGCCTGCAAGCTTCT
	Rev	GGTGGAACGGCTGACAGCTA
RASSF1	For	AGGTGAACTTGCAATGCGC
	Rev	ACCTCTGTGGCGACTTCATCT
GAPDH	For	TGCACCACCAACTGCTTA
	Rev	AGAGGCAGGGATGATGTTC
**Gene Name**		**Sequences used for Pyrosequencing (5'→ 3')**
SPARC	For	TTTTTGAGGTGGGTTGTTTTGATTAA
	Rev	Biotin/TACCCTCTAAACAAAAAAAAAACTATTCT
	Seq	ATTTGTTTAGGGGTTGTTG
FABP7	For	TAGGTATAAGGGTTGTAGTGTGAG
	Rev	Biotin/TATCCCTCTTTCCAAAAAACTATCACAA
	Seq	AAGAGGATTGGAGTTTTA
SOX4	For	AGGGATTAAGTGTTAGAGATTATGT
	Rev	Biotin/TTTCCCTAAAACAATTAATTCCAATTCAC
	Seq	AGATTATGTAGTTTTTTTGAGTTAT
MUC1	For	ATTTTTGGGTAGGGTATAAGGG
	Rev	Biotin/CAAAAACCCCAAATTCCAAACTAC
	Seq	GGTAGGGTATAAGGGTTTTA
HAS2	For	Biotin/GTTTGTAGGAGTTGGAAGTTTAGATTG
	Rev	CCACCCCTTCCCTTCTTTTTTTC
	Seq	ATAAAAAAAAAAACTAAAATAACC
HAS3	For	GTGAAAAAGAAGAGGAGGAATTGT
	Rev	Biotin/AAACCAAAACAACAAAACCTTCCTACT
	Seq	GGAATTGTTTTGGTTTTAAGA

BUN: Blood urea nitrogen; ALT: Alanine aminotransferase; AST: Aspartate aminotransferase; CK: Creatinine kinase.

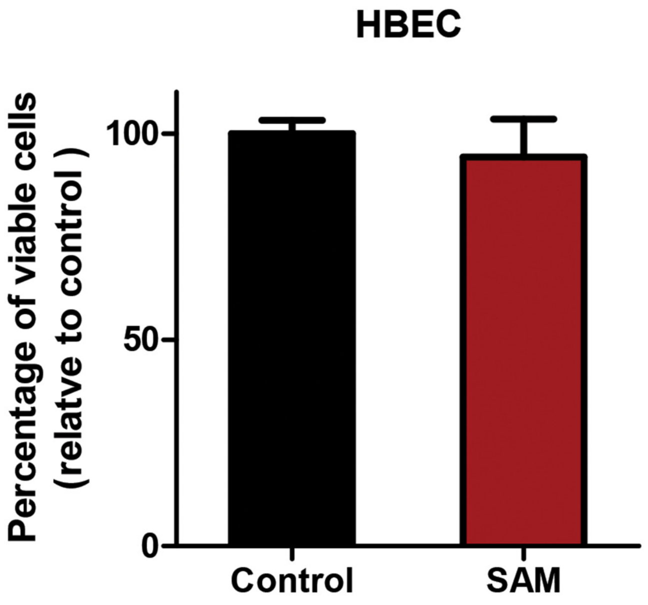

Supplementary Figure 1: Effect of SAM-treatment on the viability of human breast epithelial cells (HBEC). Briefly, HBEC were plated at the same density and treated with vehicle only control or 200 μM SAM every other day from day 2 until they were harvested on day 7. The cells were trypsinized, stained with trypan blue and the number of viable cells was counted under a light microscope daily throughout the 6-day treatments. Results from triplicate experiments are shown as a bar graph.

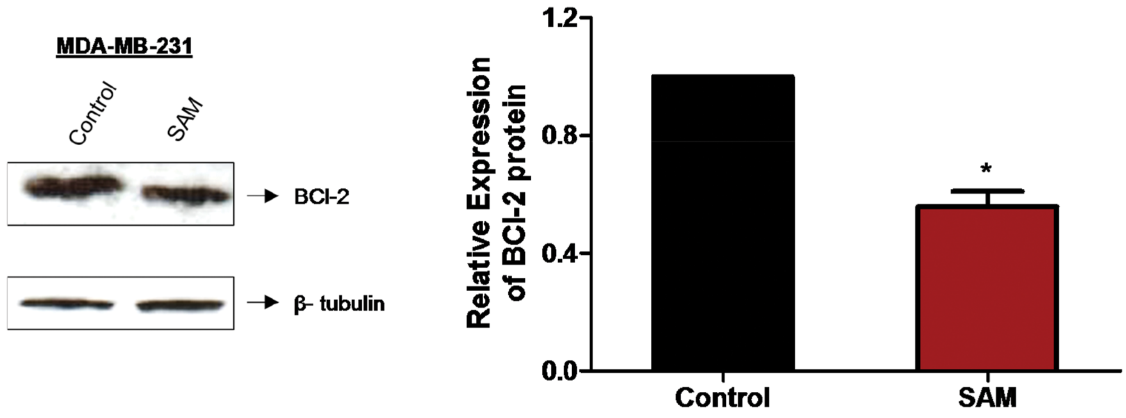

Supplementary Figure 2: Western blot from the whole cell lysates of control and 200 µM SAM-treated MDA-MB-231 cells. The membrane was probed with mouse monoclonal anti-BCl-2 (Santa Cruz Biotechnology, Cat#sc-7382) antibody. β- tubulin (BD Pharmingen, Cat#556321) was used as a loading control. The densitometric intensities of the bands were quantified by ImageJ (Fiji plugin) and plotted as bar graph in the right panel. Treatment with SAM caused a reduction in the expression of anti-apoptotic protein BCl-2 suggesting that SAM induces apoptosis by reducing the level of BCl-2. Results are shown as mean ± SEM of three independent experiments. ($^{*}P < 0.05$).

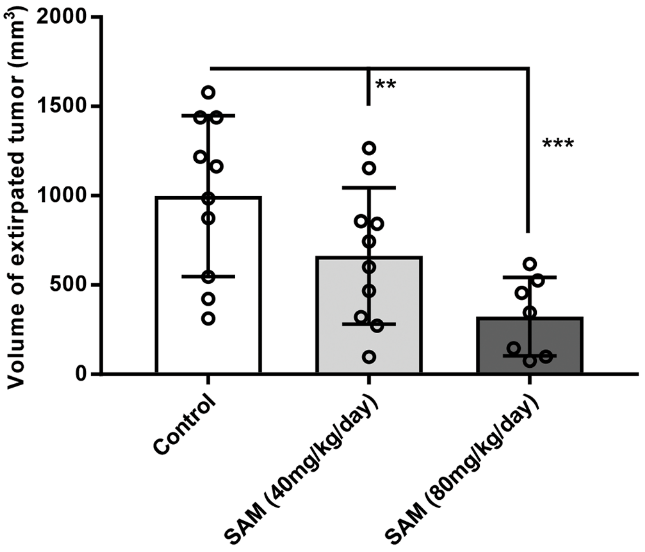

Supplementary Figure 3: The volume of the extracted mammary tumor measured after sacrificing the animal from different groups. Results are shown as mean ± SEM of at least seven animals in each group. Significant differences were determined using ANOVA followed by *post hoc* Bonferroni test and are represented by asterisks ($^{**}P < 0.01$; $^{***}P < 0.001$).

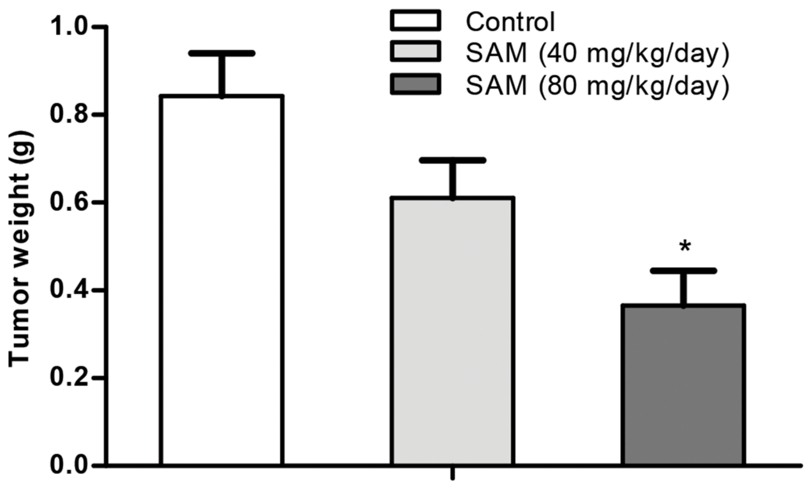

Supplementary Figure 4: Tumor weight after sacrifice. The weight of the extracted mammary tumor measured after sacrificing the animal from different groups on week 10. There is a dose-dependent reduction in tumor weight upon SAM-treatment. Results are shown as mean ± SEM of at least seven animals in each group. Significant differences are represented by asterisks ($^{*}P < 0.05$).

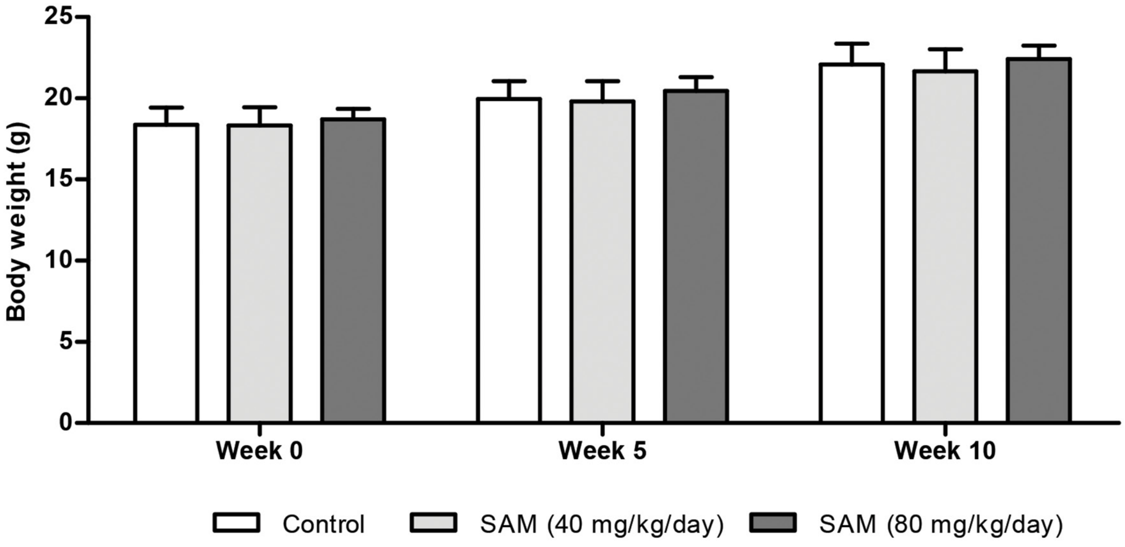

Supplementary Figure 5: Body weight of the xenograft mice. The total body weight of control and SAM-treated animals measured at different time points from the beginning of the study when the tumor cells were injected into the fourth mammary fad of the immunocompromised mice on week 0 until they were sacrificed on week 10. There was no significant difference in the weight of the animals.

A

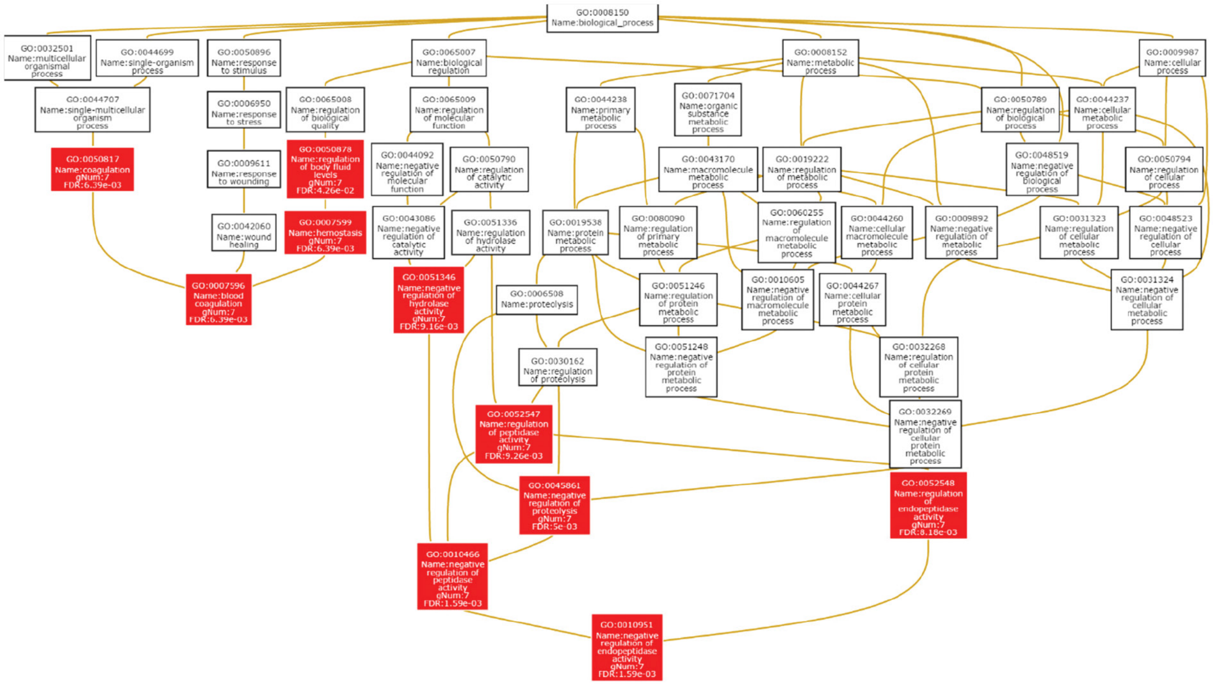

B

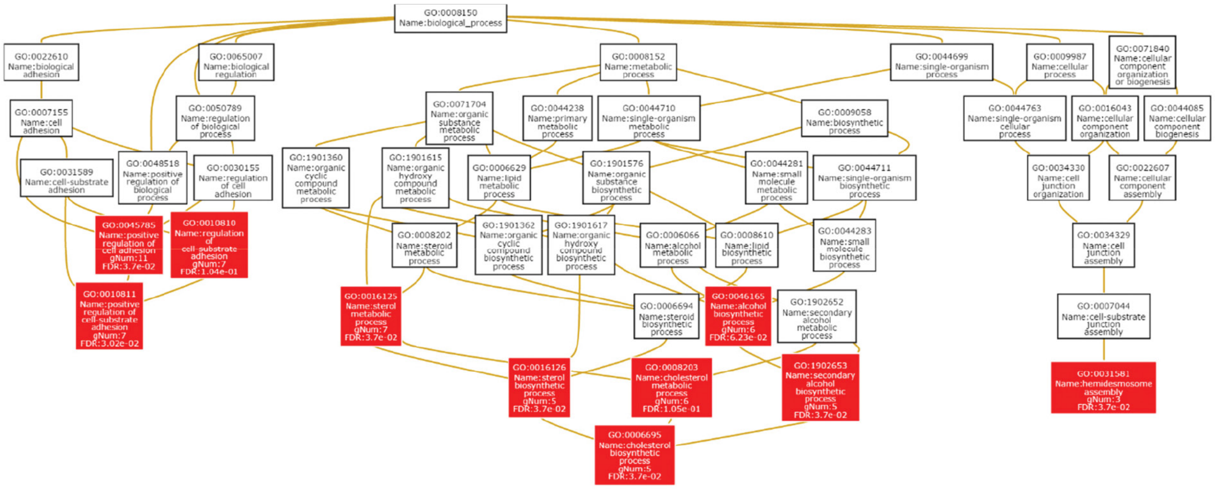

Supplementary Figure 6: Gene Ontology (GO) analysis of the top 10 biological processes enriched in the genes that upregulated **(A)** and downregulated **(B)** by SAM-treatment using WebGestalt.

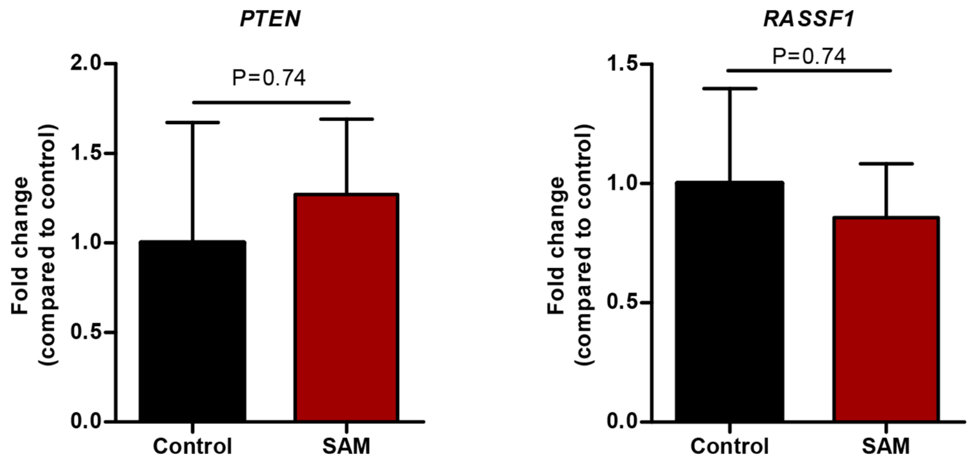

Supplementary Figure 7: Measurement of the gene expression of tumor suppressor genes. Briefly, qPCR for two well-known tumor suppressor genes (*PTEN, RASSF1*) from the RNA isolated from tumor samples showed no significant difference between control and high dose SAM (80 mg/kg/day). The results are shown are mean ± SEM from three different mice in each group.

Supplementary Table 1: SAM-treatment is non-toxic *in vivo* as shown by different parameters tested from the blood sample obtained from control and 80 mg/kg/day SAM-treated mice

Parameter	Control mice	SAM-treated mice	*P*-value
Total protein (g/L)	38.67 ± 1.19	36.33 ± 0.54	0.22
Albumin (g/L)	19.33 ± 0.72	17.67 ± 0.27	0.15
Albumin/Globulin ratio	0.97 ± 0.03	0.93 ± 0.03	0.52
Glucose (mmol/L)	14.13 ± 0.94	15.10 ± 0.86	0.57
BUN Urea (mmol/L)	8.27 ± 0.50	10.67 ± 0.59	0.06
Creatinine (µmol/L)	9.33 ± 0.27	9.0 ± 0.82	0.77
Total Bilirubin (µmol/L)	4.0 ± 0.47	5.67 ± 0.27	0.07
ALT (U/L)	37.0 ± 1.69	38.33 ± 1.96	0.69
AST (U/L)	65.33 ± 12.95	65.33 ± 6.83	1.0
CK (U/L)	105.33 ± 47.65	74.0 ± 17.21	0.64
Sodium (mmol/L)	148.67 ± 0.72	145.33 ± 0.72	0.06
Potassium (mmol/L)	4.60 ± 0.24	4.50 ± 0.22	0.77
Chloride (mmol/L)	108.33 ± 1.19	106.67 ± 0.98	0.43
Calcium (mmol/L)	2.26 ± 0.05	2.35 ± 0.04	0.22
Magnesium (mmol/L)	1.04 ± 0.01	1.67 ± 0.04	0.08

The results are shown are mean±SEM from three different mice in each group. BUN: Blood urea nitrogen; ALT: Alanine aminotransferase; AST: Aspartate aminotransferase; CK: Creatinine kinase

Supplementary Table 2: Differentially expressed mRNA probes upon SAM-treatment

Supplementary File 1:
